# The characteristics and risk factors of human papillomavirus infection: an outpatient population-based study in Changsha, Hunan

**DOI:** 10.1038/s41598-021-94635-1

**Published:** 2021-07-23

**Authors:** Bingsi Gao, Yu-Ligh Liou, Yang Yu, Lingxiao Zou, Waixing Li, Huan Huang, Aiqian Zhang, Dabao Xu, Xingping Zhao

**Affiliations:** 1 Department of Obstetrics and Gynecology, The Third Xiangya Hospital of Central South University, Changsha, 410013 China; 2 Xiangya Medical Laboratory, Central South University, Changsha, 410078 China

**Keywords:** Cervical cancer, Cancer epidemiology

## Abstract

This cross-sectional study investigated the characteristics of cervical HPV infection in Changsha area and explored the influence of *Candida* vaginitis on this infection. From 11 August 2017 to 11 September 2018, 12,628 outpatient participants ranged from 19 to 84 years old were enrolled and analyzed. HPV DNA was amplified and tested by HPV GenoArray Test Kit. The vaginal ecology was detected by microscopic and biochemistry examinations. The diagnosis of *Candida* vaginitis was based on microscopic examination (spores, and/or hypha) and biochemical testing (galactosidase) for vaginal discharge by experts. Statistical analyses were performed using SAS 9.4. Continuous and categorical variables were analyzed by t-tests and by Chi-square tests, respectively. HPV infection risk factors were analyzed using multivariate logistic regression. Of the total number of participants, 1753 were infected with HPV (13.88%). Females aged ≥ 40 to < 50 years constituted the largest population of HPV-infected females (31.26%). The top 5 HPV subtypes affecting this population of 1753 infected females were the following: HPV-52 (28.01%), HPV-58 (14.83%), CP8304 (11.47%), HPV-53 (10.84%), and HPV-39 (9.64%). Age (OR 1.01; 95% CI 1–1.01; P < 0.05) and alcohol consumption (OR 1.30; 95% CI 1.09–1.56; P < 0.01) were found to be risk factors for HPV infection. However, the presence of *Candida* in the vaginal flora was found to be a protective factor against HPV infection (OR 0.62; 95% CI 0.48–0.8; P < 0.001). Comparing with our previous study of 2016, we conclude that the subtype distribution of HPV infection is relatively constant in Changsha. Our data suggest a negative correlation between vaginal *Candida* and HPV, however, more radical HPV management is required in this area for perimenopausal women and those who regularly consume alcohol.

## Introduction

Human papillomavirus (HPV) infection is the most prevalent sexually transmitted infection in women. Persistent infection with high-risk HPV (hrHPV) subtypes, but not all HPV infections, can lead to the development of cervical cancer (CC), a disease that adversely affects the health of women worldwide^1^. Nearly 989,000 new cases of CC are diagnosed in China every year, and the overall hrHPV infection rate in mainland Chinese women is 19.0% (95% confidence intervals [CI] 17.1–20.9%)^2^. The top 5 hrHPV subtypes with the highest infection rates in China include HPV-16, HPV-52, HPV-58, HPV-53, and HPV-18^3^. HPV infection is most prevalent among childbearing-age and menopausal women; the prevalence among menopausal women is known as the “second-peak”^4^. Some of the factors reported as closely correlated with HPV infection include alcohol consumption, smoking, age at first marriage, marital status, vulvovaginal ulcers, and vulvovaginal inflammation^5,6^. The biological mechanisms of risk factors on HPV infection are less well understood over the decades. However, these investigations have yielded few results when it comes to the pathogenesis of this infection.

In recent years, scientists have become interested in and made promising progress investigating the mechanisms of vaginal flora. Some studies have reported that vaginal microorganisms might promote or impede HPV infection by infecting a host’s cervical micro-ecological environment, and may further play essential roles in the pathogenicity of HPV and cervical lesions^7^. *Lactobacillus* maintains a low vaginal pH value through producing lactic acid to perform HPV protection^8^, and *Candida* stimulates T cell proliferation to against HPV infection^9^. However, *Gardnerella*, *Fusobacteria*, *Mycobiota*, and *Chlamydial trachomatis* (CT) were associated with HPV infection^8,10,11^, for example, CT plays as the entryway of the cervical epithelium and facilitate HPV infection^11^.

In 2016, Xiao et al. reported on the characteristics of HPV infection in Changsha based on information obtained from the Gynecological Outpatient Center of our hospital (January 2009–December 2013, The Third Xiangya Hospital of Central South University)^12^. However, more evidence and investigation need to be updated to analyze the longitude development of cervical cancer prevention and treatment in Changsha. Many risk factors of cervical cancer include lifestyle such as smoking, HPV type and distribution, and the influence of vaginal flora on HPV need to be updated. We examined patients that attended the Physical Examination Outpatients Center at our hospital and hypothesized that the distribution of HPV might have changed in the few years since this previous study. We also hypothesized that vaginal flora or *Candida* might play a vital role in influencing this infection.

## Results

### Population baseline characteristics

Of the 12,628 enrolled female participants, from 19 to 84 years old, 10,875 were HPV negative and 1753 were HPV positive; the overall infection rate was 13.88% (1753/12,628). There were significant differences between the HPV-negative and -positive participants in mean age (42.57 ± 9.99, 43.83 ± 10.49 correspondingly, P < 0.001), vaginal pH (4.35 ± 0.25, 4.37 ± 0.25, separately, P < 0.001), vaginal samples positive for galactosidase (P < 0.001), sialidase, leukocyte esterase (P = 0.001), and *Candida* infection (P < 0.001), age at first marriage (P < 0.001), age at first childbirth (P < 0.001), and alcohol consumption (P = 0.003). Additionally, there were no significant differences between the two groups in BMI (P = 0.855), white blood cell (WBC) count (P = 0.873), neutrophil percentage (P = 0.298), lymphocyte percentage (P = 0.199), fasting blood glucose (P = 0.199), vaginal H_2_O_2_ (P = 0.199), vaginal grades (P = 0.227), vaginal *trichomonas* (P = 0.482), age of menstruation onset (P = 0.874), whether they had given birth (P = 0.106), diet [including spicy diet (P = 0.224) and sweet diet (P = 0.716)], smoking (P = 0.299), daily sitting time (P = 0.748), and waistline measurements (P = 0.704) (Table 1).

**Table 1:** Baseline characteristics of the study population.

	Non-HPV-infected N = 10,875	HPV-infected N = 1753	P Value
Age (year)	42.57 (9.99)	43.83 (10.49)	< 0.001
BMI (weight/height^2^)	22.64 (2.82)	22.62 (2.83)	0.855
WBC count (× 1000/mm^3^)	6.10 (1.53)	6.10 (1.51)	0.873
Neutrophil percentage	59.32 (8.09)	59.54 (7.95)	0.298
Lymphocyte percentage	32.76 (7.54)	32.51 (7.44)	0.199
Fasting blood glucose (mmol/L)	5.29 (0.83)	5.33 (0.96)	0.101
Vaginal microecology—pH	4.35 (0.25)	4.37 (0.25)	< 0.001
	**n (%)**		
**Vaginal microecology—galactosidase% (n/N)**			< 0.001
Negative	10,061 (92.5)	1669 (95.2)	
Positive	814 (7.5)	84 (4.8)	
**Vaginal microecology—sialidase% (n/N)**			< 0.001
Negative	10,388 (95.5)	1629 (92.9)	
Positive	487 (4.5)	124 (7.1)	
**Vaginal microecology—leukocyte esterase% (n/N)**			0.001
Negative	8898 (81.8)	1373 (78.3)	
Positive	1977 (18.2)	380 (21.7)	
**Vaginal microecology—H**_**2**_**O**_**2**_ **% (n/N)**			0.899
Negative	129 (1.2)	22 (1.3)	
Positive	10,746 (98.8)	1731 (98.7)	
**Vaginal microecology—*Candida* infection% (n/N)**			< 0.001
Negative	10,103 (92.9)	1678 (95.7)	
Positive	772 (7.1)	75 (4.3)	
**Vaginal microecology—*Trichomonas*% (n/N)**			0.482
Negative	10,835 (99.6)	1744 (99.5)	
Positive	40 (0.4)	9 (0.5)	
**Vaginal microecology—Grades% (n/N)**			0.277
I–II	8317 (76.5)	1325 (75.6)	
III	2547 (23.4)	428 (24.4)	
IV	11 (0.1)	0 (0.0)	
**Age of menstruation onset (y)**			0.874
< 12	1983 (18.2)	323 (18.4)	
≥ 12	8892 (81.8)	1430 (81.6)	
**Age at first marriage (y)**			< 0.001
< 20	895 (8.2)	191 (10.9)	
≥ 20	9702 (89.2)	1503 (85.7)	
NK	278 (2.6)	59 (3.4)	
**Gave birth**			0.106
Yes	623 (5.7)	91 (5.2)	
No	9974 (91.7)	1603 (91.4)	
**Age at first childbirth (y)**			< 0.001
< 20	356 (3.3)	85 (4.8)	
≥ 20 to < 30	8165 (75.1)	1347 (76.8)	
> 30	1457 (13.4)	171 (9.8)	
**Salty diet**			0.227
Less salty	5348 (49.2)	845 (48.2)	
More salty	2545 (23.4)	443 (25.3)	
**Smoking**			0.299
Never	10,333 (95.0)	1668 (95.2)	
Rarely	349 (3.2)	50 (2.9)	
Quit	29 (0.3)	9 (0.5)	
Daily	164 (1.5)	26 (1.5)	
**Alcohol consumption**			0.003
No/quit	10,009 (92.0)	1576 (89.9)	
Yes (> once a week)	866 (8.0)	177 (10.1)	
**Daily sitting time (excluding work and study)**			0.748
< 2 h	3170 (29.1)	510 (29.1)	
2–4 h	4445 (40.9)	708 (40.4)	
4–6 h	2095 (19.3)	332 (18.9)	
> 6 h	1165 (10.7)	203 (11.6)	
**Spicy diet**			0.224
Yes	4064 (37.4)	628 (35.8)	
No	6811 (62.6)	1125 (64.2)	
**Sweet diet**			0.716
Yes	2645 (24.3)	434 (24.8)	
No	8230 (75.7)	1319 (75.2)	
**Waistline**			0.704
Normal	8342 (76.7)	1349 (77.0)	
Abnormal	1967 (18.1)	321 (18.3)	

The mean and standard error are being presented for continuous variables. Continuous variables were analyzed using t-tests, and categorical variables were analyzed using chi-square tests.

*y* years, *h* hours, *BMI* body mass index, *WBC* white blood cells.

Normal Male Waistline = height (cm)/2–11 (cm).

Normal Female Waistline = height (cm)/2–14 (cm) ± 5%.

Age, BMI, WBC, neutrophil percentage, lymphocyte percentage, fasting glucose, vaginal microecology were presented as mean ± SD (standard deviation).

Grade Ι: Clean vision, there are large numbers of vaginal *Lactobacillus* and epithelial cells, no bacteria or WBCs, normal secretions.

Grade II: The amount of vaginal *Lactobacillus* and epithelial cells, a small number of WBCs and other bacteria are still normal vaginal secretions.

Grade III: A little vaginal *Lactobacillus* and squamous epithelium, more bacteria, and WBCs, suggesting a milder vaginal inflammation.

Grade IV: No vaginal Lactobacillus, only a few epithelial cells, large numbers of WBCs and miscellaneous bacteria.

The average characteristic values for 12,628 participants were calculated. This number was reached after excluding some potential participants: 4077 due to missing vaginal microecology; 2 outliers in vaginal cleanliness as measured by vaginal microecology (shown as "+" and "−", respectively); 4 due to missing information as to age of menstruation onset; 637 due to missing height information; 639 due to missing weight information; and 7 due to missing fasting blood glucose information.

### Age distribution of HPV-positive participants

We analyzed the age distribution of the study participants. The highest positive infection rate of HPV was 31.26% in 40–49.9 years old group (n = 548; 4.34% of total numbers), followed by 27.44% in 30–39.9 years old group (n = 481), and 24.59% in 50–59.9 years old group (n = 431). This data showed the HPV distribution of different age groups (Table 2).

**Table 2:** Age distribution of HPV-infected participants.

Age (y)	Participants	≥ 18 to < 20	≥ 20 to < 30	≥ 30 to < 40	≥ 40 to < 50	≥ 50 to < 60	≥ 60 to < 70	≥ 70
HPV-infected (n)		3	156	481	548	431	129	5
Of infected (%)	Infected (1753)	0.171	8.899	27.439	31.261	24.586	7.359	0.285

The inclusion age of this study is ≥ 18.

### HPV subtype distribution

**Figure 1:**
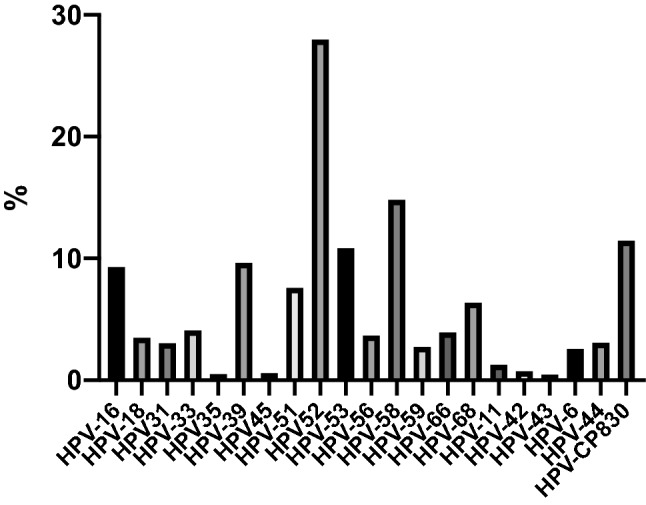
Subtype distribution in 1753 HPV-infected participants.

The percentage of multiple HPV infection was observed in the study. In the study, the top 5 HPV subtypes were HPV-52 (n = 491; 28.01%), HPV-58 (n = 260; 14.83%), CP8304 (n = 201; 11.47%), HPV-53 (n = 190; 10.84%), and HPV-39 (n = 169; 9.64%), respectively. These results are strongly consistent with previous research performed at our hospital^12^ (Table 3, Fig. 1).

**Table 3:** Subtype distribution in 1753 HPV-infected participants.

HPV subtypes	%(n/N)	n
HPV-16	9.30	163
HPV-18	3.48	61
HPV-31	3.02	53
HPV-33	2.40	42
HPV-35	0.51	9
HPV-39	9.64	169
HPV-45	0.91	16
HPV-51	7.59	133
HPV-52	28.01	491
HPV-53	10.84	190
HPV-56	3.65	64
HPV-58	14.83	260
HPV-59	2.68	47
HPV-66	3.94	69
HPV-68	5.36	94
HPV-11	1.25	22
HPV-42	0.74	13
HPV-43	0.46	8
HPV-6	2.57	45
HPV-44	3.08	54
HPV-CP8304	11.47	201

### Risk and protective factors of HPV infection identified using multiple logistic regression analysis

The trends of the unadjusted OR (OR^u^) and the adjusted OR (OR^a^) were found to be consistent. The risk factors of HPV infection were age (OR^a^ 1.01; 95% CI 1–1.01; P = 0.011) and alcohol consumption (OR^a^ 1.31; 95% CI 1.09–1.56; P < 0.01). However, vaginal *Candida* infections (OR^a^ 0.62; 95% CI 0.48–0.8; P < 0.001), age at first marriage (≥ 20 years, OR^a^ 0.79; 95% CI 0.65–0.95; P = 0.012), and age at first childbirth (> 30 years, OR^a^ 0.67; 95% CI 0.49–0.93; P = 0.016) were protective factors against HPV infection in the study. Additionally, the age at first childbirth ≥ 20 to < 30 years (OR^a^ 0.88; 95% CI 0.67–1.17) was also found to be protective against HPV infection, but the difference was not significant (P = 0.38) (Table 4).

**Table 4:** Risk and protective factors of HPV infection by the corresponding 95% confidence intervals.

	Odds ratio^u^	95% CI^u^	Odds ratio^a^	95% CI^a^	*P*-value
Age	1.01	1.01–1.02	1.01	1–1.01	0.011
**Vaginal microecology *Candida* infection**					
Negative	1.00	Reference	1.00	Reference	
Positive	0.58	0.46–0.75	0.62	0.48–0.8	< 0.001
**Age at first marriage (y)**					
< 20	1.00	Reference	1.00	Reference	
≥ 20	0.73	0.62–0.86	0.79	0.65–0.95	0.012
**Age at first childbirth (y)**					
< 20	1.00	Reference	1.00	Reference	
≥ 20 to < 30	0.69	0.54–0.88	0.88	0.67–1.17	0.38
> 30	0.49	0.37–0.65	0.67	0.49–0.93	0.016
**Alcohol consumption**					
No/quit	1.00	Reference	1.00	Reference	
Yes (> 1/week)	1.30	1.09–1.54	1.31	1.09–1.56	< 0.01

Odds ratio^u^ (OR^u^) is the unadjusted OR value; odds ratio^a^ (OR^a^) is the adjusted OR value; 95% CI^u^ are the unadjusted CI; 95% CI^a^ are the adjusted CI.

### Assessment of prediction accuracy using tenfold cross-validation

**Figure 2:**
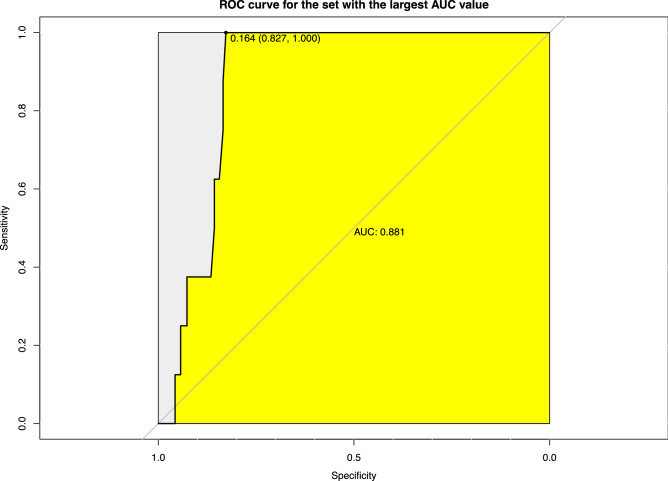
The ROC curve of HPV risk prediction logistic regression model with fungal infection. Using tenfold cross-validation, AUC values were calculated for the ROC curves of significant variables. The largest AUC as a predictive factor for HPV infection was that of fungal infection. The AUC value was 0.881, the optimal cutoff value was 0.164, the sensitivity was 1.000, and the specificity was 0.827.

The AUCs (area under curves) were significant at vaginal *Candida* infection, age at first sexual intercourse, age at first childbirth, and alcohol consumption sectors in the study (P < 0.05). The AUC values for all these variables were found to be approximately 0.8. Additionally, the ANOVA test and the initial models that included all variables produced results that were not significant, which indicates that the statistics using AUC were reliable. As an example, the largest AUC as a predictive factor for HPV infection was that of vaginal *Candida* infection. This AUC value was 0.881, the optimal cut off value was 0.164, the sensitivity was 1.000, and the specificity was 0.827 (Fig. 2).

## Discussion

The overall HPV-positive rate of this study was lower (13.88%) than that of the overall in mainland Chinese women (19.0%)^3^. In our study, the top five HPV subtypes were the following: HPV-52, HPV-58, HPV-CP8304, HPV-53, and HPV-39. However, the top 5 subtypes found in the study of Xiao et al., performed at the same hospital as our study, were HPV-52, HPV-16, HPV-58, HPV-CP8304, and HPV-53^12^, and the top 5 HPV subtypes found in China overall were HPV-16, HPV-52, HPV-58, HPV-53, and HPV-18^3^. The detection results of our hospital in recent five years showed that the positive rates of hrHPV and HPV16 were lower than the past and the national average level, indicating that Changsha city has achieved good results in reducing the high risk of HPV16 and related cervical precancerous lesions. The HPV-CP8304 is a popular subtype in our region with high prevalence but low risk. Our data were predominately consistent with the previous investigation^12^ and mostly in line with the analysis of national data^3^. These differences may be a result of variable geography, ethnicity, education, and horizontal health care. However, when considering vertical health care, this decrease in HPV infection evident in our study may be explained by improvements in women's health care and the improvements in awareness of women's health in recent years^13,14^.

Persistent hrHPV infection was believed as the causative agent in over 90% CC in early in 2000 by Bosch et al.^15,16^. In 2015, Wang et al., confirmed that the hrHPV rate was 91.8% patients with invasive cervical cancer (ICC) in Hunan province^17^. Also, HPV-16 and HPV-18 are considered the top hrHPV subtypes worldwide^18–20^, However, neither HPV-16 (6th) nor HPV-18 was identified as a prevalent subtype in our study. Several factors may account for this, such as regional differences, improvements in women's health care, and the awareness of women's health. Another reason is the participants involvement differences between studies. We investigated women from the Physical Examination Outpatient Center from our study while others from the Gynecological Outpatient Center. Symptomatic or sick patients would generally choose the Gynecological Outpatient Center for CC screening, while the Physical Examination Outpatient Center encounters more asymptomatic or healthy cases. Taking this into account, although our data is reasonably consistent with previous studies, the differences were unavoidable. These screening sample differences also suggest that the CC screening at physical examination outpatient centers, rather than the gynecological outpatient centers, might receive a closer result compared to the rate of HPV infection across the whole country.

The age distribution was later investigated. The age group with the highest HPV infection rate was 40–50 years old, followed 30–40 years, then 50–60 years. This finding is not in a full agreement with the report of the bimodal pattern in the distribution of HPV infection by age, with peaks at 26–30 years and 46–50 years^21^. Several factors might contribute to this pattern, including that immunity decreases with age, meaning that older women are at an increased the risk of developing HPV infections^22^. Besides, the postmenopausal population have elevated pH values due to the decreased estrogen levels that is correlated to the low glycogen levels and *Lactobacillus* abundance, making them sensitive to HPV infection^23,24^. Additionally, older women tend to seek out for routine gynecological care and cancer screenings^25^, which potentially elevates the HPV-positive rate for this age group. This explains our finding that increased age may be a risk factor for HPV infection (OR 1.01, 95% CI 1–1.01, P < 0.05) (Table 4). In young women, however, the risk of developing HPV infection might be more associated with an active sex life and less awareness of sexual protection^6^. There were some limitations on our research with regard to participants, including limited cases of HPV infection and lack of information regarding participants’ marital status^26^, number of sexual partners^4^, education levels, living conditions, and their use of oral contraceptives^27^. However, our findings still suggest that more attention should be paid to aged women and more routine gynecological examinations should be provided as part of routine health care for women of senior populations.

We also investigated potential risk factors of HPV infection. Our data showed that the vaginal pH of the HPV-positive group was significantly elevated than that of the HPV-negative group (4.37 ± 0.25, 4.35 ± 0.25, separately, P < 0.001), this might due to the vaginal dysbiosis, and particularly with the displacement of *Lactobacillus*^28^. Besides, the sialidase was positively correlated with HPV infection (4.5%, 487/10,875; 7.1%, 124/1753, separately, P < 0.001). This might be explained that the sialidase, one of the virulent biomarkers of *Gardneralla* vaginalis, that through hindering the epithelial biofilm formation, facilitates the infection/co-infection of HPV and other microorganisms (such as bacterial communities, chlamydial, virus such as human immunodeficiency virus (HIV), herpes simplex virus (HSV), human cytomegalovirus (HCMV)^29–35^. In line with the study of Lili et al., that the leukocyte esterase positive rate is higher in the HPV-positive group when compared with that of the HPV-negative (21.7%, 380/1753; 18.2%, 1977/10,857, separately, P = 0.001)^36^. We then figured out that alcohol consumption was correlated with HPV infection. An underlying reason for this could be that alcohol may increase sexual disinhibition, which results in increased unsafe sexual behaviors^37,38^. Smoking is another potential risk factor. Multiple studies have posited that both passive and active smoking of cigarettes increase the risk of developing HPV infection, as smoking suppresses innate immunity and can cause structural and functional changes within the respiratory system^39–41^. However, our data found no significant correlation between smoking and HPV infection. This is possibly due to limited case numbers of HPV-infected participants and the relatively restricted region within which participants lived.

Meanwhile, the probable protective factors were summarized. Our study found that a marriage age of 20 years or older (OR 0.79) and an age at first childbirth of 30 years or older (OR 0.67) were protective against HPV infection (P < 0.05). Consistent with these findings, Niyazi et al. reported that early marriage might be a risk factor for hrHPV infection^6^. This phenomenon might be explained by the notion that older women are more likely to engage in safe sexual behaviors^6^. Additionally, older women are believed to have greater awareness of genital hygiene and healthcare, which reduces the rate of HPV infection^42^. The vaginal *Candida* infection was significantly negatively correlated with HPV infection (OR 0.62; 95% CI 0.48–0.8; P < 0.001). This might be due to the presenting symptoms of *Candida* vaginitis, such as leukorrhea, vulvar pruritus, dyspareunia, and dysuria^43^, encouraging patients to seek timely gynecological screening. Additionally, *Candida parapsilosis* can serve as a biofilm on the surface of the genital tract, which may act as a shield against invasion by other microorganisms^44^. Liang et al. also supports this result; they reported similar findings of *Candida* albicans being protective factor against HPV infection (OR 0.63, 95% CI 0.49–0.82, P < 0.05)^45^. Engberts et al. also reported that infection by *Candida* does not increase the risk of developing CC^46^. Additionally, Gonia et al. found that *Candida parapsilosis* protects premature intestinal epithelial cells from invasion and damage by other microorganisms^47^. Wang et al. also reported that *Candida* albicans can enhance T cell proliferation, which might play a vital role in regulating the local vaginal and cervical microenvironments, and further inhibit the pathogenicity of HPV infection and cervical lesions^9^. However, the mechanisms that mediate the relationship between *Candida* infections and HPV infection remain controversial. Studies of larger sample sizes and further investigations are required to explore these mechanisms.

Some deficiencies cannot be ignored for this study. Besides the limited number of HPV cases and lack of information regarding participants’ marital status, number of sexual partners, use of oral contraceptives, and the history of sexual transmitted infections (STIs) such as *Gonorrhea*, *Syphilis* and HIV that might infect the vaginal-cervical microbiota^48,49^. There are further constraints in our study that might limit its wider applicability. We neither investigate details of alcohol consumption, nor follow up on infection persistence, progression, or the outcomes of participants. Besides, our documented vaginal microecology narrowly focused on *Candida* of different fugal types, more preliminary clinical findings need to be further studied, with the rest of vaginal cofounders, such as *Gardnerella*, *Fusobacterium*, bacterial vaginosis and aerobic vaginitis^8,10,45,50^. Furthermore, we excluded the previous positive HPV patients with or without local drug administration which might bias its distribution. Additionally, we did not further investigate the participants infected by multiple subtypes of HPV.

Despite these limitations, our study is in strong agreement with previous research. Additionally, this study is the first to explore the potential relationship between vaginal *Candida* with HPV infection in Changsha, Hunan. Our findings potentially provide valuable information to assist in the improvement of clinical HPV screening and CC prevention in local regions. To improve future research, larger sample sizes, optimization of the participant questionnaire, improved follow-up of participants, and in vitro experimentation might be considered.

In conclusion, we found that the prevalence of HPV infection and the distribution of its subtypes is relatively constant in Changsha, Hunan. Our data suggest that vaginal *Candida* infection is a protective factor against HPV infection and that more radical HPV management is required in the local Changsha area for perimenopausal women and who regularly consume alcohol.

## Materials and methods

### Participants

Every female patient that attended the Physical Examination Outpatients Center at the Third Xiangya Hospital of Central South University between 11 August 2017 and 11 September 2018 was asked to complete a questionnaire and voluntarily sign written informed consent. This study was approved by the ethics committee of the Third Xiangya Hospital of Central South University (IRB No. 20017). All methods were carried out in accordance with relevant guidelines and regulations.

To be included in this study, participants had to satisfy all of the following inclusion criteria: (1) age ≥ 18 years old and no previous positive HPV results; (2) no sexual intercourse, vaginal douching, or administration of vaginal medications in the 3 days before vaginal samples taken; (3) mentally competent so as to understand the consent form and communicate with study staff. The exclusion criteria: (1) history of no sexual activity; (2) HPV positive results in one year or a history of CC; (3) received a hysterectomy; (4) received treatment for vaginitis in the past 3 months; (5) had sexual intercourse, vaginal douching, or taken vaginal medication in the 3 days before vaginal samples taken; (6) unable understand the consent form and communicate with study staff; (7) the pregnant; (8) withdraw from the study.

### Medical data and diagnosis information

Patient data extracted from medical records, including general information (age, body mass index [BMI], waist measurements, age of menstruation onset, age at first marriage, childbirth history, age at first childbirth, eating habits [salty diet: less salty, more salty; spicy diet: yes, no; sweet diet: yes/no], lifestyle habits [smoking: ever, rarely, quit or daily; alcohol consumption: no/yes; daily sitting time: < 2 h, 2–4 h, 4–6 h or > 6 h]) and test results (key peripheral blood test results which were automatically calculated and generated by machine (WBC count, neutrophil percentage, lymphocyte percentage, fasting blood glucose) and vaginal microecology test results which were diagnoses by experienced cytologist (galactosidase-negative/positive; sialidase-negative/positive; leukocyte esterase-negative/positive; H_2_O_2_-negative/positive; *Candida*-negative/positive; *Trichomonas*-negative/positive; grades-I/II/III/IV (Grade I was dominated by *Lactobacillus* species. Grade II represents an intermediate status between grade I and grade III, with the presence of *L. iners, L. gasseri*, *L. crispatus, Atopobium vaginae*, *Gardnerella vaginalis*, *Actinomyces neuii* and *Peptoniphilus*. Grade III is characterized by the presence of BV-associated species (*Prevotella bivia*, *A. vaginae*, *G. vaginalis*, *Bacteroides ureolyticus* and *Mobiluncus curtisii)* and low amounts of *Lactobacillus* species, mainly *L. iners*. Finally, Grade IV is characterized by the presence of a variety of *Streptococcus* spp.)^51,52^ were carefully recorded and double-checked by two research assistants.

The vaginal and cervical samples were collected by gynecologist, while the blood was drawn by nurse. All vaginal samples were collected for the test of vaginal microecology, the cervical samples were collected for HPV screening, and the blood samples were collected for lab testing. The Pentaplex Vaginitis Detection kit (Rhfay, Guangzhou) was used for vaginal ecology testing, including galactosidase, sialidase, leukocyte esterase, H_2_O_2_, and pH value. The diagnosis of *Candida* vaginitis is based on microscopic examination (spores, and/or hypha) and biochemical testing (galactosidase) for vaginal discharge by experts. In our study, the criteria of bacterial infection diagnosis by Amsel method are as follows: (1) uniform vaginal secretion; (2) pH > 4.5; (3) amine smell in secretion with 10% potassium hydroxide; (4) positive laboratory test results (Gram staining for bacteria in secretion or wet film for clue cells). If any three of the above criteria are met (but the last one is necessary), the diagnosis can be made.

A total of 12,628 participants with complete medical records were enrolled in this study and retrospectively analyzed; 10,875 were non-HPV infected females and 1753 were HPV-infected females (Supplementary Information).

### Human papillomavirus typing

HPV DNA was amplified by polymerase chain reaction (PCR). Then, HPV genotyping by HybriMax was performed using an HPV GenoArray Test Kit (HybriBio Ltd., Chaozhou, China). This assay can determine 21 HPV types, including 14 high-risk HPV types (16, 18, 31, 33, 35, 39, 45, 51, 52, 56, 58, 59, 66, and 68), five low-risk HPV types (6, 11, 42, 43, and 44), and two unknown-risk types (53 and CP8304), by the flow-through hybridization technique using HPV DNA amplified by PCR^53^.

### Statistical analysis

Statistical analyses were performed using Statistical Analysis System 9.4 (SAS Institute, USA). Continuous variables were analyzed by t-tests, and the categorical variables were analyzed by Chi-square tests. The age distribution and subtypes distribution were summarized based on HPV positive cases.

The multivariate logistic regression risk model was used for investigating the risk and protective factors of HPV infection. The risk factor was defined as odds ratio (OR) > 1 and the protective factor was OR < 1. The multivariable regression model was established in three steps. Firstly, univariate analyses were performed to demonstrate which patient variables correlated with the presence of HPV infection with a significance of P < 0.05, and the analysis of variance (ANOVA) test and all variables were significantly different in the model (P < 0.05). Next, non-significant variables (P > 0.05) were removed, and stepwise regression was performed using the forward and backward method. Finally, the variables demonstrated as significant (P < 0.05), including age, a vaginal microecology test sample positive for fungal infection, age at first sexual intercourse, age at first childbirth, and alcohol consumption were included in the model. Analyses were performed using tenfold cross-validation, that the data set is divided into 10 parts, including 9 parts as training data and 1 part as test data. The final data is generated area under the curve (AUC) values of the receiver operating characteristic (ROC) curves. This was then used to determine the model's classification ability, and AUCs were compared to assess prediction accuracy. A P value of < 0.05 was considered statistically significant.

### Ethical statement

The authors are accountable for all aspects of the work in ensuring that questions related to the accuracy or integrity of any part of the work are appropriately investigated and resolved.

## Supplementary Information

Supplementary Information.

## Abbreviations

HPV: Human papillomavirus
hrHPV: High-risk HPV
CC: Cervical cancer
ICC: Invasive cervical cancer
95% [CI]: 95% Confidence intervals
BMI: Body mass index
PCR: Polymerase chain reaction
AUC: Area under the curve
ROC: Receiver operating characteristic
WBC: White blood cell
OR: Odds ratio
CT: *Chlamydial trachomatis*
HIV: Human immunodeficiency virus
HSV: Herpes simplex virus
HCMV: Human cytomegalovirus

## References

[1] Munoz N (2000). Human papillomavirus and cancer: The epidemiological evidence. J. Clin. Virol..

[2] Chen W, Zheng R, Baade PD, Zhang S, Zeng H, Bray F (2016). Cancer statistics in China, 2015. CA Cancer J. Clin..

[3] Li K, Li Q, Song L, Wang D, Yin R (2019). The distribution and prevalence of human papillomavirus in women in mainland China. Cancer.

[4] Brogaard KA, Munk C, Iftner T, Frederiksen K, Kjaer SK (2014). Detection of oncogenic genital human papillomavirus (HPV) among HPV negative older and younger women after 7 years of follow-up. J. Med. Virol..

[5] Bell MC, Schmidt-Grimminger D, Jacobsen C, Chauhan SC, Maher DM, Buchwald DS (2011). Risk factors for HPV infection among American Indian and white women in the Northern Plains. Gynecol. Oncol..

[6] Niyazi M, Husaiyin S, Han L, Mamat H, Husaiyin K, Wang L (2016). Prevalence of and risk factors for high-risk human papillomavirus infection: A population-based study from Hetian, Xinjiang, China. Bosn. J. Basic Med. Sci..

[7] Zheng JJ, Song JH, Yu CX, Wang F, Wang PC, Meng JW (2019). Difference in vaginal microecology, local immunity and HPV infection among childbearing-age women with different degrees of cervical lesions in Inner Mongolia. BMC Womens Health..

[8] Lee JE, Lee S, Lee H, Song YM, Lee K, Han MJ (2013). Association of the vaginal microbiota with human papillomavirus infection in a Korean twin cohort. PLoS One..

[9] Wang X, Coleman HN, Nagarajan U, Spencer HJ, Nakagawa M (2013). Candida skin test reagent as a novel adjuvant for a human papillomavirus peptide-based therapeutic vaccine. Vaccine..

[10] Murta EF, Souza MA, Araujo Junior E, Adad SJ (2000). Incidence of *Gardnerella vaginalis*, *Candida* sp. and human papilloma virus in cytological smears. Sao Paulo Med. J..

[11] Silva J, Cerqueira F, Medeiros R (2014). Chlamydia trachomatis infection: Implications for HPV status and cervical cancer. Arch. Gynecol. Obstet..

[12] Xiao SS, Fan JL, He SL, Li YR, Wang LY, Yu KN (2016). Analysis of human papillomavirus infection in 16,320 patients from a gynecology clinic in Central South China. J. Low Genit. Tract. Dis..

[13] Li J, Kang J, Mao Y, Zheng P, Abdullah AS, Wu G (2020). Investigating HPV- and HPV vaccine-related knowledge, perceptions, and information sources among health care providers in three big cities in China. Vaccines (Basel)..

[14] You D, Han L, Li L, Hu J, Zimet GD, Alias H (2020). Human papillomavirus (HPV) vaccine uptake and the willingness to receive the HPV vaccination among female college students in China: A multicenter study. Vaccines (Basel)..

[15] Melnikow J, Henderson JT, Burda BU, Senger CA, Durbin S, Weyrich MS (2018). Screening for cervical cancer with high-risk human papillomavirus testing: Updated evidence report and systematic review for the US preventive services task force. JAMA.

[16] Bosch FX, Lorincz A, Munoz N, Meijer CJ, Shah KV (2002). The causal relation between human papillomavirus and cervical cancer. J. Clin. Pathol..

[17] Wang L, Wu B, Li J, Chen L (2015). Prevalence of human papillomavirus and its genotype among 1336 invasive cervical cancer patients in Hunan province, central south China. J. Med. Virol..

[18] Li K, Yin R, Li Q, Wang D (2017). Analysis of HPV distribution in patients with cervical precancerous lesions in Western China. Medicine (Baltimore).

[19] Parkin DM, Louie KS, Clifford G (2008). Burden and trends of type-specific human papillomavirus infections and related diseases in the Asia Pacific region. Vaccine..

[20] Vinodhini K, Shanmughapriya S, Das BC, Natarajaseenivasan K (2012). Prevalence and risk factors of HPV infection among women from various provinces of the world. Arch. Gynecol. Obstet..

[21] Chan PK, Chang AR, Yu MY, Li WH, Chan MY, Yeung AC (2010). Age distribution of human papillomavirus infection and cervical neoplasia reflects caveats of cervical screening policies. Int. J. Cancer..

[22] Dartell M, Rasch V, Munk C, Kahesa C, Mwaiselage J, Iftner T (2013). Risk factors for high-risk human papillomavirus detection among HIV-negative and HIV-positive women from Tanzania. Sex Transm. Dis..

[23] Straight SW, Herman B, McCance DJ (1995). The E5 oncoprotein of human papillomavirus type 16 inhibits the acidification of endosomes in human keratinocytes. J. Virol..

[24] Brotman RM, Shardell MD, Gajer P, Tracy JK, Zenilman JM, Ravel J (2014). Interplay between the temporal dynamics of the vaginal microbiota and human papillomavirus detection. J. Infect. Dis..

[25] Smith EM, Ritchie JM, Levy BT, Zhang W, Wang D, Haugen TH (2003). Prevalence and persistence of human papillomavirus in postmenopausal age women. Cancer Detect. Prev..

[26] Kero KM, Rautava J, Syrjanen K, Kortekangas-Savolainen O, Grenman S, Syrjanen S (2014). Stable marital relationship protects men from oral and genital HPV infections. Eur. J. Clin. Microbiol. Infect. Dis..

[27] Sreedevi A, Javed R, Dinesh A (2015). Epidemiology of cervical cancer with special focus on India. Int. J. Womens Health..

[28] van de Wijgert JH, Borgdorff H, Verhelst R, Crucitti T, Francis S, Verstraelen H (2014). The vaginal microbiota: What have we learned after a decade of molecular characterization?. PLoS One..

[29] Guo YL, You K, Qiao J, Zhao YM, Geng L (2012). Bacterial vaginosis is conducive to the persistence of HPV infection. Int. J. STD AIDS..

[30] Onywera H, Williamson AL, Mbulawa ZZA, Coetzee D, Meiring TL (2019). Factors associated with the composition and diversity of the cervical microbiota of reproductive-age Black South African women: A retrospective cross-sectional study. PeerJ.

[31] Santiago GL, Deschaght P, El Aila N, Kiama TN, Verstraelen H, Jefferson KK (2011). *Gardnerella vaginalis* comprises three distinct genotypes of which only two produce sialidase. Am. J. Obstet. Gynecol..

[32] Di Paola M, Sani C, Clemente AM, Iossa A, Perissi E, Castronovo G (2017). Characterization of cervico-vaginal microbiota in women developing persistent high-risk Human Papillomavirus infection. Sci. Rep..

[33] Alves P, Castro J, Sousa C, Cereija TB, Cerca N (2014). *Gardnerella vaginalis* outcompetes 29 other bacterial species isolated from patients with bacterial vaginosis, using in an in vitro biofilm formation model. J. Infect. Dis..

[34] Guidry JT, Scott RS (2017). The interaction between human papillomavirus and other viruses. Virus Res..

[35] Di Pietro M, Filardo S, Porpora MG, Recine N, Latino MA, Sessa R (2018). HPV/Chlamydia trachomatis co-infection: Metagenomic analysis of cervical microbiota in asymptomatic women. New Microbiol..

[36] Li L, Ding L, Gao T, Lyu Y, Wang M, Song L (2020). Association between vaginal micro-environment disorder and cervical intraepithelial neoplasia in a community based population in China. J. Cancer..

[37] Nyitray AG, da Silva RJ, Baggio ML, Lu B, Smith D, Abrahamsen M (2011). The prevalence of genital HPV and factors associated with oncogenic HPV among men having sex with men and men having sex with women and men: the HIM study. Sex Transm. Dis..

[38] Tolstrup J, Munk C, Thomsen BL, Svare E, van den Brule AJ, Gronbaek M (2006). The role of smoking and alcohol intake in the development of high-grade squamous intraepithelial lesions among high-risk HPV-positive women. Acta Obstet. Gynecol. Scand..

[39] Feng RM, Hu SY, Zhao FH, Zhang R, Zhang X, Wallach AI (2017). Role of active and passive smoking in high-risk human papillomavirus infection and cervical intraepithelial neoplasia grade 2 or worse. J. Gynecol. Oncol..

[40] Kumar R, Rai AK, Das D, Das R, Kumar RS, Sarma A (2015). Alcohol and tobacco increases risk of high risk HPV infection in head and neck cancer patients: Study from North-East Region of India. PLoS One..

[41] Mehta H, Nazzal K, Sadikot RT (2008). Cigarette smoking and innate immunity. Inflamm. Res..

[42] Smith EM, Johnson SR, Ritchie JM, Feddersen D, Wang D, Turek LP (2004). Persistent HPV infection in postmenopausal age women. Int. J. Gynaecol. Obstet..

[43] Mtibaa L, Fakhfakh N, Kallel A, Belhadj S, Belhaj Salah N, Bada N (2017). Vulvovaginal candidiasis: Etiology, symptomatology and risk factors. J. Mycol. Med..

[44] Kuhn DM, George T, Chandra J, Mukherjee PK, Ghannoum MA (2002). Antifungal susceptibility of Candida biofilms: Unique efficacy of amphotericin B lipid formulations and echinocandins. Antimicrob. Agents Chemother..

[45] Liang Y, Chen M, Qin L, Wan B, Wang H (2019). A meta-analysis of the relationship between vaginal microecology, human papillomavirus infection and cervical intraepithelial neoplasia. Infect. Agent Cancer..

[46] Engberts MK, Vermeulen CF, Verbruggen BS, van Haaften M, Boon ME, Heintz AP (2006). Candida and squamous (pre)neoplasia of immigrants and Dutch women as established in population-based cervical screening. Int. J. Gynecol. Cancer..

[47] Gonia S, Archambault L, Shevik M, Altendahl M, Fellows E, Bliss JM (2017). *Candida parapsilosis* protects premature intestinal epithelial cells from invasion and damage by *Candida albicans*. Front. Pediatr..

[48] Lacey CJ (2019). HPV vaccination in HIV infection. Papillomavirus Res..

[49] Gatechompol S, Teeratakulpisarn N, Wittawatmongkol O, Teeraananchai S, Kerr SJ, Chalermchockcharoenkit A (2020). Incidence, persistence, and factors associated with HPV infection among male adolescents with and without perinatally acquired HIV infection. J. Acquir. Immune Defic. Syndr..

[50] Sodhani P, Gupta S, Gupta R, Mehrotra R (2017). Bacterial vaginosis and cervical intraepithelial neoplasia: Is there an association or is co-existence incidental?. Asian Pac. J. Cancer Prev..

[51] Ravel J, Gajer P, Abdo Z, Schneider GM, Koenig SS, McCulle SL (2011). Vaginal microbiome of reproductive-age women. Proc. Natl. Acad. Sci. U. S. A..

[52] Petrova MI, Lievens E, Malik S, Imholz N, Lebeer S (2015). Lactobacillus species as biomarkers and agents that can promote various aspects of vaginal health. Front. Physiol..

[53] Tao P, Zheng W, Wang Y, Bian ML (2012). Sensitive HPV genotyping based on the flow-through hybridization and gene chip. J. Biomed. Biotechnol..

