# Supplementary material for: The characteristics and risk factors of human papillomavirus infection: an outpatient population-based study in Changsha, Hunan
Source: Sci Rep. 2021 Jul 23;11:15128. doi: 10.1038/s41598-021-94635-1 (PMC8302608; doi:10.1038/s41598-021-94635-1)
Supplement: Supplementary file 1 — Supplementary Information. [file 41598_2021_94635_MOESM1_ESM.docx]

Supplementary table: Calculating data of Table 1

|  | **0** | **1** | **p** | **test** |
| --- | --- | --- | --- | --- |
| n | 10875 | 1753 |  |  |
| age..mean..sd.. | 42.57 (9.99) | 43.83 (10.49) | <0.001 |  |
| WCcount..mean..sd.. | 6.10 (1.53) | 6.10 (1.51) | 0.873 |  |
| Neutrophil...mean..sd.. | 59.32 (8.09) | 59.54 (7.95) | 0.298 |  |
| lym...mean..sd.. | 32.76 (7.54) | 32.51 (7.44) | 0.199 |  |
| GLU0..mean..sd.. | 5.29 (0.83) | 5.33 (0.96) | 0.101 |  |
| tctmould...1.... | 216 (2.0) | 41 (2.3) | 0.379 |  |
| tcttrichomonad...1.... | 50 (0.5) | 17 (1.0) | 0.011 |  |
| tct_herpesvirus...0.... | 10875 (100.0) | 1753 (100.0) | NA |  |
| tctsum.... |  |  | <0.001 |  |
| X...0 | 7231 (66.5) | 818 (46.7) |  |  |
| X...1 | 3583 (32.9) | 689 (39.3) |  |  |
| X...2 | 55 (0.5) | 176 (10.0) |  |  |
| X...3 | 6 (0.1) | 70 (4.0) |  |  |
| vaginagalac...1.... | 814 (7.5) | 84 (4.8) | <0.001 |  |
| vaginasialidase...1.... | 487 (4.5) | 124 (7.1) | <0.001 |  |
| viaginaleukesterase...1.... | 1977 (18.2) | 380 (21.7) | 0.001 |  |
| viaginaH2O2...1.... | 10746 (98.8) | 1731 (98.7) | 0.899 |  |
| viaginapH..mean..sd.. | 4.35 (0.25) | 4.37 (0.25) | <0.001 |  |
| viaginamould...1.... | 772 (7.1) | 75 (4.3) | <0.001 |  |
| viaginatrichomonad...1.... | 40 (0.4) | 9 (0.5) | 0.482 |  |
| viaginaclear.... |  |  | 0.277 |  |
| X...2.1 | 8317 (76.5) | 1325 (75.6) |  |  |
| X...3.1 | 2547 (23.4) | 428 (24.4) |  |  |
| X...4 | 11 (0.1) | 0 (0.0) |  |  |
| firstperiod...2.... | 8892 (81.8) | 1430 (81.6) | 0.874 |  |
| firstsexlife.... |  |  | <0.001 |  |
| X...1.1 | 895 (8.2) | 191 (10.9) |  |  |
| X...2.2 | 9702 (89.2) | 1503 (85.7) |  |  |
| X...99 | 278 (2.6) | 59 (3.4) |  |  |
| givebirth.... |  |  | 0.106 |  |
| X...0.1 | 623 (5.7) | 91 (5.2) |  |  |
| X...1.2 | 9974 (91.7) | 1603 (91.4) |  |  |
| X...99.1 | 278 (2.6) | 59 (3.4) |  |  |
| firstgivebirthage.... |  |  | <0.001 |  |
| X...1.3 | 356 (3.3) | 85 (4.8) |  |  |
| X...2.3 | 8165 (75.1) | 1347 (76.8) |  |  |
| X...3.2 | 1457 (13.4) | 171 (9.8) |  |  |
| X...99.2 | 897 (8.2) | 150 (8.6) |  |  |
| appetite.... |  |  | 0.227 |  |
| X...1.4 | 5348 (49.2) | 845 (48.2) |  |  |
| X...2.4 | 2545 (23.4) | 443 (25.3) |  |  |
| X...3.3 | 2982 (27.4) | 465 (26.5) |  |  |
| smoking.... |  |  | 0.299 |  |
| X...0.2 | 10333 (95.0) | 1668 (95.2) |  |  |
| X...1.5 | 349 (3.2) | 50 (2.9) |  |  |
| X...2.5 | 29 (0.3) | 9 (0.5) |  |  |
| X...3.4 | 164 (1.5) | 26 (1.5) |  |  |
| drinking...1.... | 866 (8.0) | 177 (10.1) | 0.003 |  |
| exstudytime.... |  |  | 0.748 |  |
| X...1.6 | 3170 (29.1) | 510 (29.1) |  |  |
| X...2.6 | 4445 (40.9) | 708 (40.4) |  |  |
| X...3.5 | 2095 (19.3) | 332 (18.9) |  |  |
| X...4.1 | 1165 (10.7) | 203 (11.6) |  |  |
| spicy...1.... | 4064 (37.4) | 628 (35.8) | 0.224 |  |
| none...1.... | 3713 (34.1) | 579 (33.0) | 0.376 |  |
| dessert...1.... | 2645 (24.3) | 434 (24.8) | 0.716 |  |
| waistyn.... |  |  | 0.704 |  |
| X...0.3 | 8342 (76.7) | 1349 (77.0) |  |  |
| X...1.7 | 1967 (18.1) | 321 (18.3) |  |  |
| X...99.3 | 566 (5.2) | 83 (4.7) |  |  |
| bmi..mean..sd.. | 22.64 (2.82) | 22.62 (2.83) | 0.855 |  |

P< 0.05, significant different.
